# Supplementary figures and images for: A large-scale transgenic RNAi screen identifies transcription factors that modulate myofiber size in Drosophila
Source: PLoS Genet. 2021 Nov 15;17(11):e1009926. doi: 10.1371/journal.pgen.1009926 (PMC8629395; doi:10.1371/journal.pgen.1009926)

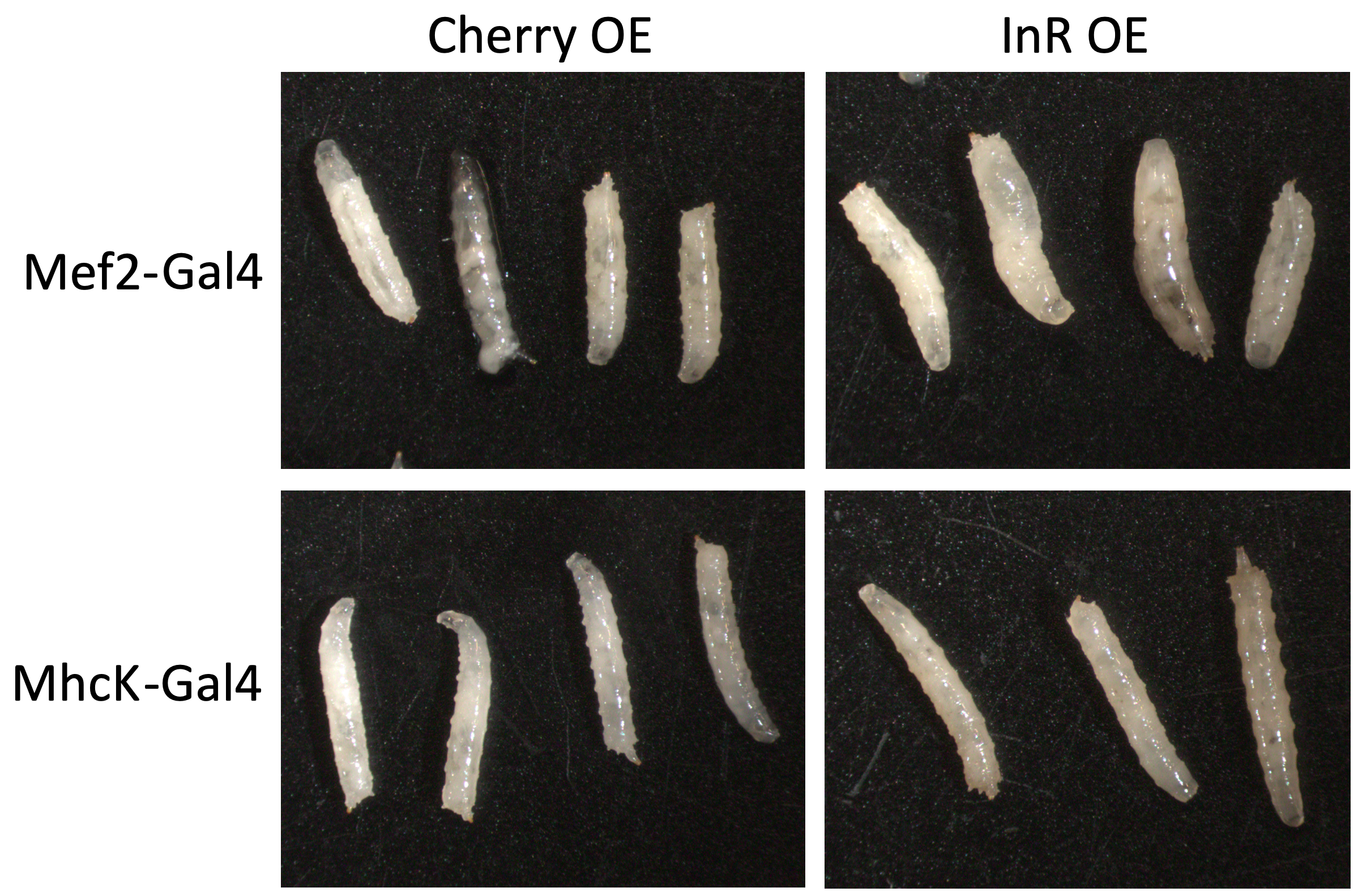

Supplement: S1 Fig — Consistent with previous studies in Drosophila and mammals, overexpression of insulin/IGF receptor (InR) in skeletal muscle via Mef2-Gal4 induces skeletal muscle hypertrophy, as indicated by the increase in body size. However, a relatively minor increase is found with InR overexpression via MhcK-Gal4 (Mhc-Gal4.K, BL#55133) suggesting that this Gal4 line is not ideal for uncovering muscle hypertrophy phenotypes. (TIF) [file pgen.1009926.s001.tif]

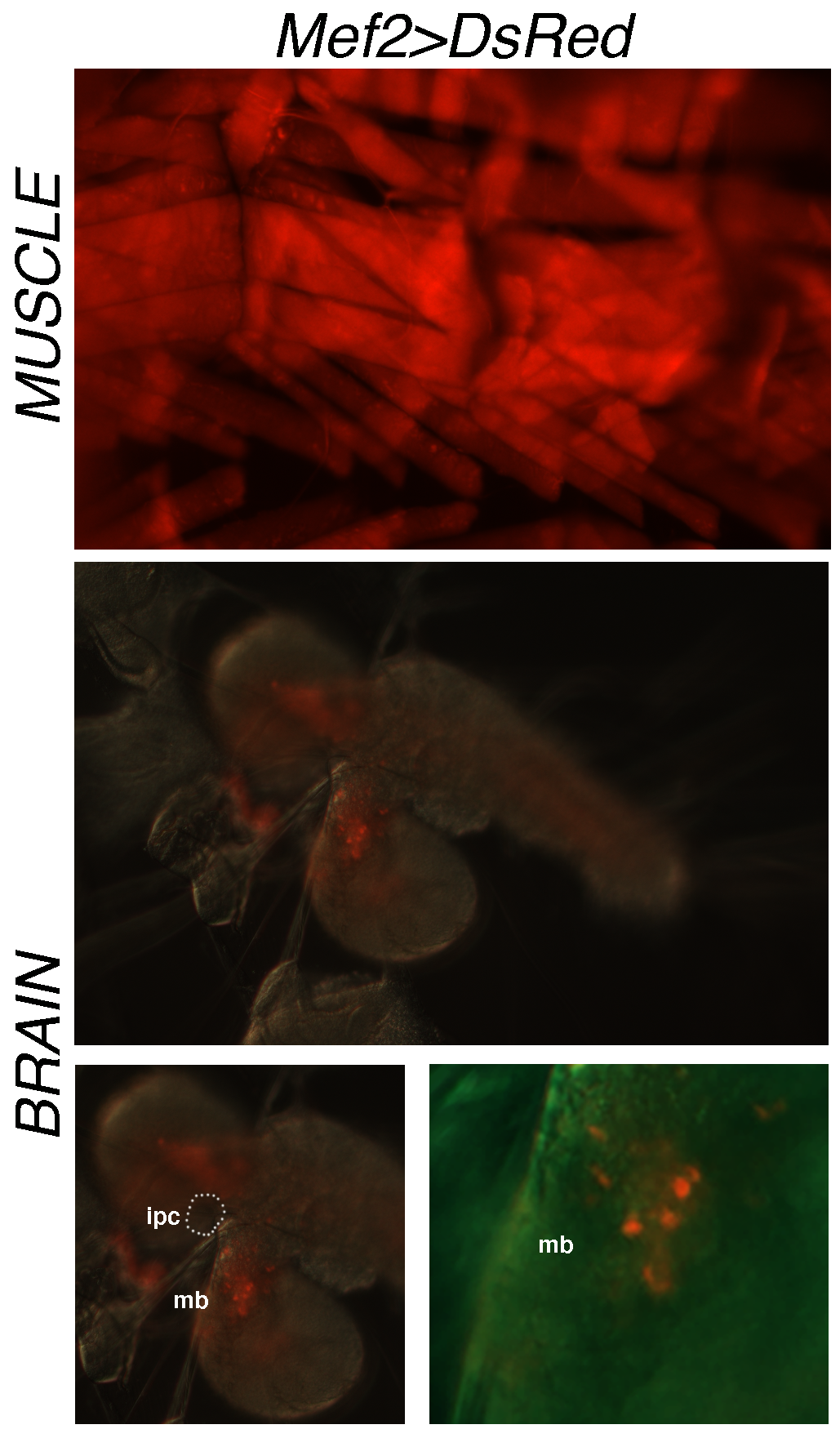

Supplement: S2 Fig — Red fluorescence due to transgenic DsRed expression is detected primarily in body wall skeletal muscles but also in visceral muscles and few cells in the brain. We find no evidence for Mef2-Gal4-driven DsRed expression in insulin producing cells (ipc) with this line. However, DsRed expression driven by Mef2-Gal4 is detected in brain cells of the mushroom body (mb), consistent with our original characterization of this driver (Demontis and Perrimon, 2009, Development; PMID:19211682) and a more recent study that has found endogenous Mef2 expression in a subset of Kenyon cells of the mushroom body (Crittenden et al. 2018, Biology Open; PMID:30115617). (TIF) [file pgen.1009926.s002.tif]

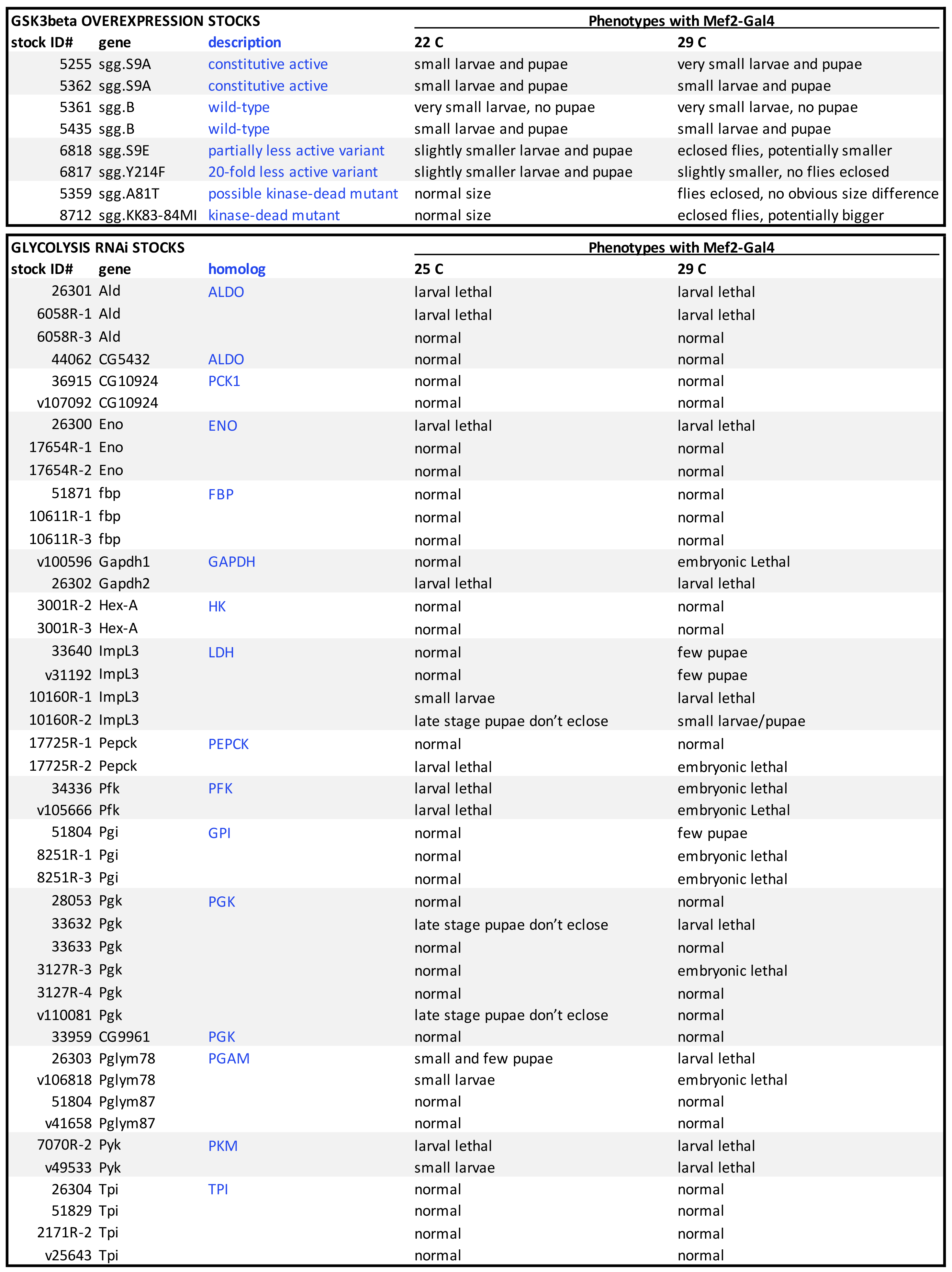

Supplement: S3 Table — (TIF) [file pgen.1009926.s005.tif]
