# Supplementary material for: A large-scale transgenic RNAi screen identifies transcription factors that modulate myofiber size in Drosophila
Source: PLoS Genet. 2021 Nov 15;17(11):e1009926. doi: 10.1371/journal.pgen.1009926 (PMC8629395; doi:10.1371/journal.pgen.1009926)
Supplement: Attachment — Submitted filename: REBUTTAL LETTER_PLoS Genetics.pdf [file pgen.1009926.s007.pdf]

REBUTTAL LETTER

Comments to the Authors:

Reviewer #1: The manuscript by Graca et al., titled A global transgenic RNAi screen identifies transcription factors that modulate myofiber size in Drosophila does most of what is suggested by the title. The authors do a fairly large and systematic RNAi screen with the goal of identifying novel transcription factors that regulate muscle growth to better understand the mechanisms that underly muscle atrophy and hypertrophy. In the opinion of this reviewer, the manuscript is exceptionally well written, provides clear and easy to interpret figures, and puts forth a mechanisms by which glycolysis is regulated for the purpose of muscle growth. Although there are some revisions that are necessary to make this manuscript suitable for publication they are relatively minor in scope.

Response: Thank you for the positive evaluation of our work. We have now addressed the points raised as explained in detail below and in the revised manuscript.

Questions and Comments

1. I personally would not use the word "global" in the title. As stated, 447 out of 708 transcription factors were investigated. Global to me suggests that all transcription factors were examined. I'd recommend "large-scale" or "systematic".

Response: We have changed the title of the manuscript accordingly to "large-scale".

2. Figure 1 and the associated methods suggest that larvae were just categorized as large, small, mishapen, or normal. Additional details indicating the threshold for variation from control necessary to categorize a larva would be helpful, particularly for readers who may try to follow up on this work.

Response: We have now indicated in the methods the reference phenotype compared to which such classification was done: "A- no larvae/no pupae; B- very small larvae/no pupae (<u>i.e. smaller than FoxO overexpression</u>); C- small larvae/small pupae (<u>i.e. similar to FoxO overexpression</u>); D- normal (no visible phenotype); E- increased larval/pupal size (<u>i.e. similar to InR overexpression</u>); and F- thin or sickle-shaped larvae with locomotor defects; J- upheld or depressed wings in the majority of flies in the tube (<u>i.e. similar to pink1 RNAi or parkin RNAi</u>)."

3. The methods associated with Figure 1 list the control as "such as white RNAi". If multiple different controls were used they should all be listed along with the rationale for using each.

Response: We have now specified in the methods that white RNAi has been used as the standard control. In some experiments (as shown in the figures), cherry RNAi was also used as additional control.

4. Figure 2 represents my biggest and most important questions regarding this manuscript. 4A. It seems that only a subset of the hits from the screen were examined in figure 2. I certainly recognize that completing this analysis of a large number of different conditions might be prohibitive. However, I think it is essential that the authors provide a rationale for choosing the hits they follow up on and ignoring the hits that they did not. If in fact all of the hits were examined, it should be stated explicitly and data for all should be provided in the supplement at the very least.

Response: We report in Figure 2 a subset of screen hits that we decided to further analyze because of their extremely high evolutionary conservation (i.e., typically, a DIOPT score >7), consistent scoring with multiple RNAi lines, and/or biological novelty (Figure 2 and Supplemental Table 1).

4B. The hemisegment chosen for muscle size analysis will impact the data. Therefore it is critical to know which hemisegments were used. Additionally, and perhaps more importantly, the hemisegments near the anterior and posterior end of the animal are significantly smaller and more variable than the central hemisegments. Based on figure 2A it seems that a subset of muscles from these hemisegments may have been used. The authors should remove these data points and rely on the data from central hemisegments.

Response: Myofiber size analysis has been done similar to our previous publications (Demontis and Perrimon, 2009; Hunt et al. 2019). Specifically, we have analyzed VL3 and VL4 muscles from hemisegment 3. We have analyzed multiple larvae for each genotype in a consistent manner and therefore are confident that the reported phenotypes are valid. However, we appreciate this feedback and will keep it in mind for future studies.

5. I hesitate to suggest this because it has the feel of making the paper what I would like it to be rather than what it is. Nevertheless, figure 5 just seems an anticlimactic way to end a very nice manuscript. It is suggestive, along with the transcriptomic data in figure 4, that Deaf1 works through regulation of glycolysis to regulate muscle growth. But, we already knew that disruption of glycolysis would impact muscle size. If the glycolysis disruption experiments could be done in the background of altered Deaf1 and/or GSK3 levels, it would be a more fitting conclusion to this manuscript. Even selecting just two glycolytic enzymes to do in this background would be a much more convincing way to show that glycolysis works downstream of Deaf1 and GSK3 to regulate muscle growth.

Response: Thank you for pointing this out. There are indeed several publications (which we have cited) about the involvement of glycolysis in muscle growth. In addition to the extensive screen via RNAi lines for glycolytic enzymes that we had previously reported, we now show in Fig. 5B the analysis of skeletal muscles with RNAi for 2 glycolytic enzymes (Eno and Pglym78). As expected based on our hypothesis and previous publications, RNAi for Eno and Pglym78 reduces the area of VL3+VL4 muscles. While we agree that it would be nice to test the functional interaction between glycolysis and Deaf1, we were not able to do so because the Deaf1 RNAi and the most effective RNAi lines for glycolytic enzymes were site-integrated on the same position in the genome (attP2 on the 3rd chromosome), preventing us from generating recombined lines. Nonetheless, our findings and previous studies collectively support the model whereby Deaf1 regulates myofiber growth at least in part via glycolysis.

Minor Comments

1. Citations vary between author, year and numbers. I presume this will be fixed and is not a concern, rather something I noticed.

Response: We have added additional citations. We apologize if we have missed any relevant ones and would be glad to include them.

2. At the bottom of page 6, the authors cite two papers as evidence that fusion is not responsible for the variations in muscle size. I do believe that this is not a fusion effect, but I do not believe that these specific disruptions were looked at in those papers. If I am correct, the authors should be clear and precise in their language.

Response: Thank you for pointing this out. We have now removed this sentence as we have not directly examined myoblast fusion in this and past work cited.

3. Regarding the statistical analysis, the authors state that all data points indicate a biological replicate. I think that this is vague and means different things to different researchers. For example, in figure 2 one might assume that each point refers to a different mating of males and virgins whereas another might think it means a different animal from a single mating. A more precise description will be helpful in understanding the variability of the data.

Response: We have now specified in the methods that each biological replicate refers to data obtained from a different larva; typically, larvae from 2 or more crosses were analyzed for each genotype. The larvae analyzed in Figures 2-5 were obtained from crosses different from those used for the screen.

4. The last sentence of the first paragraph on page 4 suggests that the sickle shape is indicative of defective contractile properties. Without something to reference, or data to support this conclusion, the authors should just report their finding.

Response: Thank you for pointing this out. We could not find a reference but anecdotally observed that these larvae move slowly. We have amended the text and removed any inference on the contractile properties as we did not investigate this phenotype.

==

Reviewer #2: The manuscript by Graca et al. reports the results of a straightforward RNAi screen in Drosophila designed to uncover roles for conserved transcription factors in muscle size control. The authors employed 1114 RNAi lines that targeted 447 transcription factors that showed some similarity to human transcription factors, for knockdown in developing muscles and then noted size defects at larval and adult stages as well as some wing positioning defects that have been noted to result from muscle abnormalities. In all about 12% of the targeted genes generated a phenotype consistent with and effect on muscle growth. The authors then performed followed up proof of principle studies on DEAf1 a conserved factor that was not known to affect muscle growth. Loss of Deaf1 was found to increase muscle size while overexpression led to smaller muscles. These phenotypes where opposite to those exhibited by manipulation of GSK3, a kinase that has been shown in mammal system to associate with and phosphorylate DEAf1. Interestingly transcriptome analysis of Deaf1 verses GSK3 knockdown and overexpression n lead to a strikingly similar regulation profile for the core genes involved in glycolysis consistent with pervious observations that inhibition of normal glycolytic flux leads to smaller muscle size. Overall, this is a short but informative communication that will provide a useful resource to those that study muscle size control in both Drosophila and mammals. In general, the experimental design and outcomes are well described and the results easy to follow. That said I think there are a few points that the authors should address before publication.

Response: Thank you for the positive evaluation of our work. We have addressed the points raised as indicated below.

1) There should be some discussion of the limitations of the screen. Obviously, there will be some false positives as well as false negatives. It is a bit hard to know what these limits are but one criterion for a true hit might be that more than one RNAi targeting a given gene gives a similar phenotype. By my count there are ~34 genes in supplement table 1 that satisfy this criterion. I think it would be useful to list these genes in a separate table within the main results and provide some limited discussion about these hits such as how many of these have been previously linked to muscle development. Likewise, it is worth pointing out that those genes with only one identified RNAI hit might simply be off target effects. Using this same data set the authors should also take the opportunity to provide an example of another type of possible false positive. I note that trachealess might be one such gene. It satisfies the two-hit criterion, but it is a big surprise, since as far as I am aware, there is no known role for trachealess in muscle development. Rather it seems to be specific for regulating genes involved in the formation of the insect oxygen delivery system. Obviously, if the mef-2 Gal4 driver has some off target expression at some stage in trachea, then it could explain the small larval size phenotype.

Response: Thank you for pointing this out. We have now better discussed the limits of our screen and what are likely to be the bona fide screen hits: a new paragraph in the discussion now reports the impact of false positives and false negatives. As requested by the reviewer, we have also re-screened the Bloomington RNAi lines for the genes that scored consistently with 2 or more RNAi lines (listed in Supplemental Table 1). This new data, which is shown in Fig. 1D-E, reports overall similar phenotypes obtained with Mef2-Gal4 and another muscle Gal4 driver, MhcK-Gal4, although there are also differences in the outcome of RNAi expression driven by these Gal4 drivers. Trachealess was identified by this reviewer as a possible false positive and indeed our re-screening with Mef2-Gal4 finds only marginal effects on larval body size (a proxy for muscle mass).

This brings up my main concern with this study which is that only one “muscle-specific” driver has been used (understandable to keep the work effort down). However, one imposed limitation of this methodology is that the results are only interpretable if the driver expression is tight with respect to the targeted tissue. Mef-2 is pretty good, but there is a big problem with some versions of this driver which is that it is also strongly expressed in the ipc neurons (at least in late third instar larvae) that produce and release several key insulin-like peptides. It is incumbent on the authors to explicitly state which mef2 driver line they are using and confirm by crossing to a UAS reporter that it is indeed muscle

specific and does not show expression in other tissues especially the IPCs. Without such confirmation, using mef-2 as the sole driver in the screen could produce false positives by alterations in insulin signaling which is a central regulator of body size through several mechanisms. Obviously, rescreening all hits with an ipc "specific driver" to rule out possible effects on insulin signaling is a large amount of work. However, if the authors Mef2 Gal4 line is expressed in IPCs, then perhaps they could just rescreen the 34 genes that satisfy the more than one RNAi hit criteria described above to determine if any of the phenotypes in this class might be caused by the off target Mef-2 expression.

Response: We have crossed UAS-DsRed with our Mef2-Gal4 line, which is the one we originally used and characterized in Demontis and Perrimon, 2009, *Development*. We find no evidence for Mef2-Gal4-driven DsRed expression in the insulin producing cells. However, transgene expression is found in brain cells of the mushroom body (Supplemental Figure S2), consistent with our original characterization of this driver (Demontis and Perrimon, 2009) and a previous report that has found endogenous expression of *Mef2* in mushroom body neurons (Crittenden et al. 2018, *Biology Open*). We recognize that this is a limitation of this tool and have therefore mentioned this in the discussion.

Concerning the use of additional tools, we have employed the Mhc-K-Gal4, which is reported to drive strong expression in skeletal muscle from embryonic development. We find that several of the screen hits that scored with 2 RNAi lines similarly score with MhcK-Gal4 as originally found for Mef2-Gal4. However, this analysis was limited to the Bloomington lines that induce atrophy because MhcK-Gal4 does not seem effective (compared to Mef2-Gal4) in uncovering muscle hypertrophy phenotypes: specifically, overexpression of insulin receptor (InR, a known induced of muscle hypertrophy in Drosophila and mammals) did not induce overt hypertrophy when driven by MhcK-Gal4 (Supplemental Figure S1), suggesting that this line may be useful for testing interventions that induce atrophy but not for uncovering hypertrophy phenotypes. (Although we do not know the reason for this, we have found in other experiments that the insertion or toxic levels of Gal4 expression can limit growth/viability of certain Gal4 lines).

2) A second issue is that the Deaf and GSK3 work leads to suggesting that they work in the same pathway but in opposite ways to modulate glycolysis. One simple test to determine their order of action is genetic epitasis. If GSK3's effect on muscle size and glycolytic enzyme expression is primarily through altering Deaf 1 activity, then muscle directed overexpression of GSK3CA together with Deaf1 RNAi should produce a Deaf1 phenotype (higher levels of glycolytic enzyme expression and larger muscle size. Have the authors tried such an experiment?

Response: Gsk3 induces atrophy via the phosphorylation of many targets, and each of these Gsk3 substrates likely contributes in part to the muscle wasting phenotype. On this basis, although the experiment here proposed is sound, inhibition of only one target (i.e. Deaf1) is unlikely to rescue Gsk3-induced muscle wasting because Gsk3 phosphorylates many target proteins that are conducive of wasting.
