# Supplementary material for: A large-scale transgenic RNAi screen identifies transcription factors that modulate myofiber size in Drosophila
Source: PLoS Genet. 2021 Nov 15;17(11):e1009926. doi: 10.1371/journal.pgen.1009926 (PMC8629395; doi:10.1371/journal.pgen.1009926)
Supplement: Attachment — Submitted filename: RESPONSE TO THE REVIEWERS.pdf [file pgen.1009926.s008.pdf]

RESPONSE TO THE REVIEWERS' COMMENTS

Comments to the Authors:
Please note here if the review is uploaded as an attachment.

Reviewer #1: The revised manuscript by Graca et al. addressed my questions and is suitable for publication with the potential of minor modifications to address one question I have.

In the last full paragraph of page 4, the authors compare the data from the Mef2 based RNAi screen to the MhcK based RNAi screen. Specifically, the similarities and the differences are highlighted. However, I question whether strict reliance on the statistical measures is appropriate here. For example, CG6724 is said to reduce larval size in Mef2 but not MhcK. But, the MhcK looks very similar to the Mef2. I do understand that the MhcK data can't be compared to the Mef2 control, but I think that this could be added to the new discussion section on the limitations of the screen. Similarly, Pc and trachealess are listed as "marginally impacted". If the authors are going to stick strictly to statistical measures, they should do so in this case also and list those RNAi lines as having no effect. Finally, the variation in the pdm3 phenotype could also be a point of discussion.

Response: Thank you for finding our manuscript suitable for publication. We have now introduced the minor modifications suggested into the revised version of the manuscript. Specifically, we have better described the data in Figure 1D-E and amended the Results section of the manuscript. The amended paragraph, with changes highlighted, is reported here below:

> Specifically, RNAi for CG7839, taf1, mtTFB2, pdm3, Su(var)3-9, and myc (dm) consistently reduced larval body area with both *Mef2-Gal4* and *MhcK-Gal4*, although pdm3 RNAi yielded stronger effects with *MhcK-Gal4* versus *Mef2-Gal4*. However, RNAi for srp and PolrMT significantly reduced larval size with *Mef2-Gal4* but not with *MhcK-Gal4*. Moreover, RNAi for CG6724 marginally reduced larval size with both *Mef2-Gal4* and *MhcK-Gal4*, although this was statistically significant only with *Mef2-Gal4.* Conversely, RNAi for Not1 reduced larval size only when driven by *MhcK-Gal4*. Lastly, RNAi for Pc and trachealess (trh), which were previously classified as screen hits, did not significantly impact larval size when rescreened with *Mef2-Gal4* (Figure 1D), suggesting that they are false positives.

Reviewer #2: The authors have done a very through job of addressing my concerns. I think it is a nice study that will be of interest to many in the muscle biology field.

Response: Thank you very much for the positive evaluation of our work.
